# Refractory obsessive-compulsive disorder: a challenging treatment

**DOI:** 10.1192/j.eurpsy.2022.752

**Published:** 2022-09-01

**Authors:** A.L. Ramos, H. Salgado

**Affiliations:** Centro Hospitalar Universitário de São João, Psiquiatria, Porto, Portugal

**Keywords:** Deep brain stimulation, transcranial direct current stimulation, obsessive-compulsive disorder, repetitive transcranial magnetic stimulation

## Abstract

**Introduction:**

Obsessive-compulsive disorder (OCD) is a chronic and impairing condition included in the DSM-5 Obsessive-Compulsive Spectrum Disorders. Despite psychopharmacological and psychotherapeutic measures, there are patients who remain refractory to different therapeutic strategies.

**Objectives:**

The authors aim to present different alternatives in approach, treatment and management of refractory OCD, based on a review of the existing literature.

**Methods:**

Analysis of the data about this subject, considering the review articles and the case reports published at current time and highlighting the most essential topics, concerning the latest developments in the area.

**Results:**

Therapeutic options are presented, including transcranial direct current stimulation (tDCS), repetitive transcranial magnetic stimulation (rTMS), deep brain stimulation (DBS) and ablative neurosurgery.

**Conclusions:**

The treatment of OCD represents a great challenge in clinical practice. Despite the advances accomplished by a more extensive knowledge of the disease and a burden of new techniques in the last decades, more treatment strategies are needed, especially for patients with non-response to conventional treatment.

**Disclosure:**

No significant relationships.

